# Temperature during pupal development affects hoverfly developmental time, adult life span, and wing length

**DOI:** 10.1002/ece3.10516

**Published:** 2023-10-24

**Authors:** Klára Daňková, Sarah Nicholas, Karin Nordström

**Affiliations:** ^1^ Flinders Health and Medical Research Institute Flinders University Adelaide South Australia Australia; ^2^ Department of Zoology, Faculty of Science Charles University Praha 2 Czech Republic; ^3^ Department of Medical Cell Biology Uppsala University Uppsala Sweden

**Keywords:** body size, Diptera, *Eristalis tenax*, locomotor activity, morphometrics, phenotypic plasticity, rearing temperature, Syrphidae, thermal performance

## Abstract

Hoverflies (Diptera, Syrphidae) are cosmopolitan, generalist flower visitors and among the most important pollinators after bees and bumblebees. The dronefly *Eristalis tenax* can be found in temperate and continental climates across the globe, often synanthropically. *Eristalis tenax* pupae of different generations and different climate zones are thus exposed to vastly different temperatures. In many insects, the ambient temperature during the pupal stage affects development, adult size, and survival; however, the effect of developmental temperature on these traits in hoverflies is comparatively poorly understood. We here reared *E*. *tenax* pupae at different temperatures, from 10°C to 25°C, and quantified the effect on adult hoverflies. We found that pupal rearing at 17°C appeared to be optimal, with high eclosion rates, longer wings, and increased adult longevity. Rearing temperatures above or below this optimum led to decreased eclosion rates, wing size, and adult survival. Similar thermal dependence has been observed in other insects. We found that rearing temperature had no significant effect on locomotor activity, coloration or weight, despite evidence of strong sexual dimorphism for each of these traits. Our findings are important as hoverflies are key pollinators, and understanding the effects of developmental temperature could potentially be useful for horticulture.

## Graphical abstract

We reared hoverfly pupae at different ambient temperatures, and measured the effect on eclosion rate, adult size and coloration, locomotor activity and survival. We found that rearing the pupae at 17°C is optimal for high eclosion rate, long life span, and larger flies.

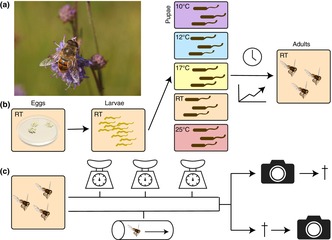

## 1 INTRODUCTION

Hoverflies are a cosmopolitan fly family with about 6000 species, containing many generalist flower visitors that feed on nectar and pollen (Klecka et al., 2018). This makes hoverflies among the most important alternative pollinators, after bees and bumblebees (Jauker et al., 2012; Rader et al., 2020). Among hoverflies, the dronefly *Eristalis tenax* is of particular agricultural interest, as it is a synanthropic species (Speight et al., 2002) found in temperate and continental climates across the globe. This includes Europe (Francuski et al., 2013), Asia (Sengupta et al., 2016), the Americas (Osburn, 1915), and Oceania (Howlett & Gee, 2019). *Eristalis* hoverflies are generalist pollinators, visiting native, wild flowers, as well as important crop plants (Jauker et al., 2009; Klecka et al., 2018; Rader et al., 2020), across a wide range of floral colors and sizes (Nordström et al., 2017). This means that *Eristalis* hoverflies are also useful as biological pollinators in, for example, commercial greenhouses (Jarlan et al., 1997).

While adult hoverflies feed from a wide array of flowers, larvae of eristaline hoverflies are aquatic/semiaquatic and thrive in organically rich rotting material (Speight, 2017). As the larvae prefer decaying organic material in stagnant water, such as manure‐polluted water around farms, *E*. *tenax* have followed human populations around the globe (Atkins, 1948). For example, they likely immigrated into western North America from Japan and Eastern Siberia, but did not spread eastwards until more intense farming practices and denser populations with associated drains and sewers became common in the 1800s (Osburn, 1915; Osten‐Sacken, 1886). Similarly, *E*. *tenax* hoverflies were introduced into New Zealand from Britain or California some time before 1888, where it is now a very abundant exotic species (see e.g., Doyle et al., 2020). Where introduced, *E*. *tenax* is common around intensely farmed agroecosystems where native pollinators do not necessarily thrive (Atkins, 1948; Stavert et al., 2018). As a consequence, dipteran larvae have been proposed as an environmentally sensible way of treating farm manure (Morales & Wolff, 2010; Roffeis et al., 2015).

As a species, *E*. *tenax* is thus found in different habitats. However, individual hoverflies may also be subjected to very different habitats and temperatures, as they are known to migrate long distances (see e.g., Doyle et al., 2020) with individually labeled hoverflies having covered hundreds of kilometers in a single day (Wotton et al., 2019). Furthermore, hoverflies in one location may be subject to different temperatures, humidity and light, since the weather can vary immensely between the first emergence of *Eristalis* in early spring and the last observation in autumn (Francuski et al., 2013; Ottenheim, 2000). The earliest females (*E*. *tenax*) that emerge in Europe in February to April are females that mated in autumn, hibernated, and then emerged to lay eggs for the new season (see Heal, 1989). This new generation of hoverflies goes on to lay eggs throughout the Northern summer.

Previous work has shown that the ambient temperature during the pupal stage affects developmental time and adult coloration in *Eristalis* hoverflies and that the effect on coloration varies greatly between species and genotypes (Heal, 1989; Ottenheim et al., 1996). Indeed, as the body temperature of ectotherms affects their physiological performance (Hochachka & Somero, 2002) and as darker animals heat up faster, the thermal melanism hypothesis predicts that exposure to lower developmental temperatures should result in darker adults (Forsman, 2011). However, how other adult characteristics, such as hoverfly survival, size, and activity, are affected by developmental temperature is less well‐understood. This is important as developmental temperature is known to have lasting effects on adult performance in a range of insects, including damselflies (Arambourou et al., 2017), mosquitoes (Ezeakacha & Yee, 2019), and dung beetles (Carter & Sheldon, 2020). For example, in over 80% of ectotherms, colder developmental temperatures lead to larger adults (Atkinson, 1994), as described by the temperature‐size rule.

The beneficial acclimation hypothesis states that prior exposure to a given temperature should optimize future performance at the same temperature (Rebolledo et al., 2021). Indeed, insect locomotion is strongly influenced not only by the ambient temperature, but also by the developmental temperature (Angilletta et al., 2002; Bahrndorff et al., 2016). More specifically, insect locomotion is impaired outside optimal temperature ranges (Berrigan & Partridge, 1997). Such temperature dependence can be described with temperature performance curves (TPCs, see e.g., Angilletta Jr., 2009), where performance increases with temperature from its lower thermal limit (*CT*_min_) to a peak (*P*_max_) at the thermal optimum (*T*_opt_), before decreasing with higher temperatures to an upper thermal limit (*CT*_max_). The shape of the curve depends on what is being quantified. For example, developmental rates can often be described with skewed TPCs (Kingsolver et al., 2011) where *T*_opt_ is closer to *CT*_max_, whereas those for survival may be more symmetrical (Van Der Have, 2002).

In order to use *Eristalis* hoverflies efficiently as pollinators of crops in fields (Cook et al., 2020; Stavert et al., 2018) or greenhouses (Garibaldi et al., 2013), and dipteran larvae for manure treatment (Bortolini et al., 2020; Roffeis et al., 2015), we need to know more about their development, and factors that affect it, such as temperature. Indeed, even if *Eristalis* hoverflies are often suggested to be commercially viable (Rader et al., 2020), efficient mass rearing is still the limiting factor (Francuski et al., 2014). Furthermore, it has been recognized that there is a need to better understand how developmental thermal stress affects adult performance (Rebolledo et al., 2021). To address this, we here expose *E*. *tenax* pupae to different temperatures, from 10°C to 25°C, and measure its effect on adult characteristics. We hypothesize that developmental rate will follow a typical TPC with a *T*_opt_ close to temperatures experienced in their native habitats and that survival will follow a similar pattern. We hypothesize that pupae reared below this *T*_opt_ will develop slower, and result in darker and larger adults. Finally, we hypothesize that animals will have higher locomotor activity in the temperature they were exposed to during development, compared with animals reared at other temperatures.

## 2 MATERIALS AND METHODS

### 2.1 Animals

**FIGURE 1:**
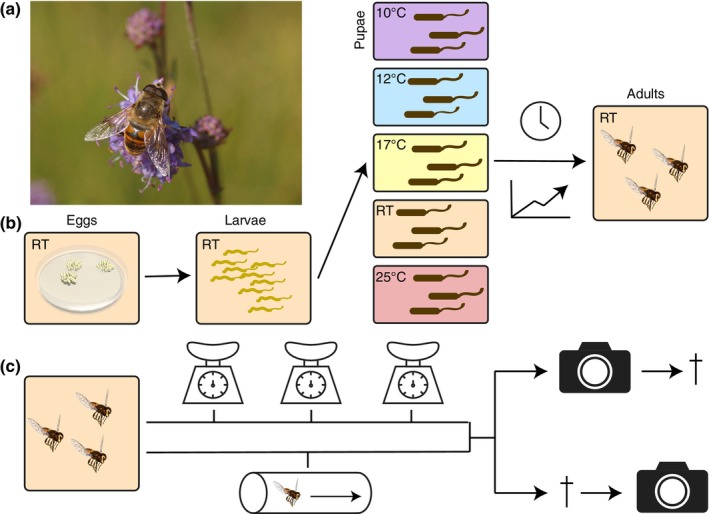
Method summary. (a) We investigated the effect of developmental temperature on the hoverfly *Eristalis tenax*. Photo by Lukáš Janošík. (b) Hoverfly eggs and larvae developed at room temperature, as described previously (Nicholas et al., 2018). Third instar larvae were separated into five different temperature regimes and kept there during the entire pupal stage. For each temperature, we measured the time until eclosion and the developmental success rate. (c) Upon eclosion, 30–40 adults from the 12°C, 17°C and 25°C rearing temperatures were kept separately and weighed every 5–15 days until the time of death. These hoverflies were not used in the analysis of locomotor activity. A total of 226 adults were photographed while still alive, and 130 after they died. The photographs were used for morphometric analysis.

We collected egg batches from female *E*. *tenax* hoverflies (Table 1) wild caught in the Wittunga Botanic Garden (Adelaide, South Australia), under a permit provided by the Department of Environment and Water (DEWR), Government of South Australia. The larvae were reared in fresh rabbit dung (Nicholas et al., 2018) at room temperature (22.8 ± 1.0°C). Each batch contained around 200 eggs (Nicholas et al., 2018). Third instar larvae were moved to small containers for rearing in different temperature conditions until the adult hoverflies emerged from the pupae (Figure 1). All pupae were kept in small, transparent containers fitted with a mesh under a 16‐h light: 8‐h dark cycle, with light provided by Arlec LED lights (UC0168, 350 lumens, warm white, Arlec Electrical Services) controlled by a timer. We exposed the pupae to five different temperatures (Figure 1b, Table 1). The coldest was achieved using a fridge (Hisense, HR6AF243, Hisense Australia) set to 10°C (10.1 ± 0.47°C). The 12°C (12.1 ± 0.54°C) and 17°C (17.4 ± 0.37°C) pupae were kept in wine coolers (Kogan 8 Bottle Thermoelectric Wine Cooler, Kogan Australia Pty Ltd). The 23°C pupae (22.8 ± 1.0°C) developed at room temperature in insect rearing cages. The 25°C (25.4 ± 1.76°C) pupae were housed on a plant propagation heating mat controlled by a THD digital controller (Aldoheat Horticultural Products). In one case, 10 pupae reared at 25°C were moved to room temperature for the last 2 days of their pupation, but treated as part of the 25°C cohort.

**TABLE 1:** Summary of egg batches used in rearing experiments.

Batch	Female ID	Pupae (*N*)	Adults (*N*)	10°C	12°C	17°C	23°C	25°C
001	W01	126	98					
003	W02‐W05	237	121					
004	W06	137	114					
005	L01‐L18	194	144					
007	L01‐L18	158	107					
010	W07	228	162					
011	L19‐L36	131	91					

*Note*: Summary of egg batches used in rearing experiments, and the pupal temperatures each batch was exposed to (filled boxes). The eggs were always sired by wild‐caught males, paired with individually labeled wild‐caught (W) or lab‐reared (L) females. When using lab‐reared females, these were generated by wild‐caught parents.

Upon eclosion, the adult flies were moved to insect rearing cages (BugDorms, Australian Entomological Supplies) with a 24.5 or 32.5 cm side for maintenance at room temperature under laboratory lights (Nicholas et al., 2018). They were fed dry pollen, sugar, and water ad libitum. When counting the eclosion rate, we treated the hoverflies that died before completely crawling out of the pupae as unsuccessful.

Adult hoverflies were labeled with unique color marks (Semco acrylic paint) on the thoracic dorsum to enable tracking of individuals. We recorded the life span of adult hoverflies from the day of eclosion to their death (Figure 1). Hoverflies that died from unnatural causes (e.g., by freezing for experimental reasons) appear as censored datapoints, and 10 individuals were omitted from the survival analysis due to lacking data about their sex.

### 2.2 Morphometrics

For the hoverflies reared at 12°C, 17°C, and 25°C, we assigned a group of individual hoverflies (30–40 per temperature, balanced sample from each sex and batch) that were kept in a separate cage and regularly weighed at an interval of 5–15 days until the time of death (Figure 1c). The first measurement took place within 1–2 weeks of emergence. Each hoverfly was weighed by placing it in a small tissue culture dish (35 × 10 mm, Sarstedt AG & Co.), using a Sauter AR 1014 electronic balance (Sauter GmbH). These measurements were taken unblinded.

After the hoverflies died, we took high‐resolution photographs of them using an Olympus SZX10 camera equipped with DF PLAPO 1X‐4 JAPAN lens (130 hoverflies, done blinded), or Olympus E‐M10 Mark II camera equipped with Olympus ED 12–50 mm f/3,5–6,3 EZ lens (57 hoverflies, unblinded). A total of 226 hoverflies were photographed while still being alive using the Olympus E‐M10 Mark II camera, and to prevent their movement during photographing, we covered them with a lid of a tissue culture dish pressed against a soft cellulose square. Camera type and status during photographing (alive/dead) were used as predictors for statistical models to filter out potential systematic variability (see Data S1). Wings were cut from living or dead specimens using scissors and were stretched with a tissue culture dish for photographing. We used a ruler to calibrate the photographs, and then extracted wing length, measured between the point where the transversal h‐vein joins the anterior edge of the wing and the point where the R4 + 5 vein joins the tip of the wing (as in Ottenheim & Volmer, 1999). We measured the length of the thorax, defined as the distance between the scutellum–metathorax border and the head–prothorax border, along the center of the metanotum. All measurements were conducted using the ImageJ software (Schneider et al., 2012).

From the photographs, we defined the coloration of tergite 2 and tergite 3, which can be yellow, orange, brown, or black (Francuski et al., 2011). We used a color scale with six steps (pictograms, Figure 6), with three lighter grades and three darker grades (as defined in Heal, 1979). For this classification, the size of the colored patches is more important than shade (Heal, 1979). However, note that this is a simplified scale as other authors have used up to 22 different *E*. *tenax* color morphs (Francuski et al., 2011).

### 2.3 Activity

To quantify locomotor activity, we used the Locomotor Activity Monitoring system (LAM25, TriKinetics Inc) with 25 mm diameter × 125 mm long Pyrex glass tubes (PGT25 × 125, TriKinetics Inc) positioned horizontally. Hoverflies were individually placed in each tube, where the ends were sealed with a cotton ball with water, pollen, and honey (Thyselius & Nordström, 2016). Additional water was added to the cotton balls twice daily (before 9:00 and after 16:00) by a Pasteur pipette to prevent drying out. Each time the hoverfly crossed the center of the tube, it would break an infrared beam, and this would be counted as an activity measurement. The hoverflies were kept in the Locomotor Activity Monitoring system (LAMS) for approximately 48 h, starting in the morning, and the average activity between 10:00 and 16:00 on the second day of recording was used for quantification. We quantified the mean activity at 12°C and 17°C by placing the LAMS in wine cooler fridges, or by placing it at room temperature, all under a 16‐h light: 8‐h dark cycle.

Sometimes a hoverfly would remain stationary in the middle of the tube for extended periods of time, continuously breaking the beam. To avoid this giving erroneously high activity measurements, all measurements indicating more than 10 crossings per minute were replaced with a 1 and all non‐null measurements immediately following it were replaced with a 0 (as in Thyselius & Nordström, 2016).

### 2.4 Statistical analysis

Throughout the paper, *N* refers to the number of flies (see Tables 1 and 2 for summary). In the text, all data are given as mean ± SD unless otherwise mentioned. Where the figures show boxplots, these indicate median and interquartile ranges, and the whiskers extend up to 1.5x of the interquartile range. Any data beyond this distance are considered as outliers, shown with individual points.

**TABLE 2:** Numbers of hoverflies used in the different analyses.

Rearing temperature	10°C	12°C	17°C	23°C	25°C
Pupal duration	17	185	167	72	123
Eclosion rate (number of batches)	104 (3)	289 (4)	218 (4)	360 (3)	240 (5)
Survival		193	159		98
Wing length		124	104		48
Thorax length		177	132		79
Weight		29	39		39
Coloration		177	132		79
Activity		79	79		65

*Note*: Number of individual hoverflies used in the statistical analyses (filled boxes). For activity measurements, some individual hoverflies were used repeatedly, but they were never used twice in the same testing temperature. For calculating eclosion rate, we used batches as the basic numerical units (given in brackets), not individual hoverflies.

All statistical analyses were performed using the R 4.2.1 software and RStudio 2022.12.0 interface (R Core Team, 2022). Pupal duration and morphometrics (wing length, thorax length, wing/thorax ratio) were analyzed by ANOVA combined with Tukey's HSD. In data with non‐normally distributed residuals, we used appropriate transformation. For count data and proportional data, we used generalized linear model (GLM) of either quasi‐poisson (over‐dispersed count data) or quasi‐binomial (proportional data) family. For ordinal response variable (coloration), we used cumulative link model (CLM). When dealing with repeated measurements (weight gain data), we checked the data using partial autocorrelation function (PACF) and found only weak temporal autocorrelation. Subsequently, we compared the results and AIC of the weight gain model with and without autocorrelation structure, and as these two reported similar results, we implemented a linear model without autocorrelation structure. Further inspection of differences in weight gain between groups was conducted using Simple Slopes Analysis (“interactions” package) and Estimated Marginal Means (“emmeans” package). For survival analysis, we used Cox proportional hazards model with time‐splitting (tt) correction. For statistical details, see Data S1 and Table 3.

**TABLE 3:** Statistical summary.

Response variable	Statistical test	Rearing temperature (°C)	Sex	Batch identity
Pupal duration	ANOVA	***	N/A	***
Eclosion rate	GLM	***	N/A	*p* = .470
Survival	Cox	***	***	***
Wing length	ANOVA	***	***	***
Thorax length	ANOVA	*p* = .191	***	***
Wing/thorax ratio	ANOVA	*p* = .541	***	*p* = .206
Weight	LM	*p* = .711	***	N/A
Coloration	CLM	*p* = .326	***	***
Activity	GLM	*p* = .405	*p* = .920	*p* = .124

*Note*: The effect of rearing temperature, sex, and batch on the different measurements done in the study. Effects of other predictors and interactions between predictors were tested in several cases (see 3. Results) but are not displayed.

*** *p* < .001 (significance code).

## 3 RESULTS

### 3.1 The rearing temperature affects hoverfly development and survival

**FIGURE 2:**
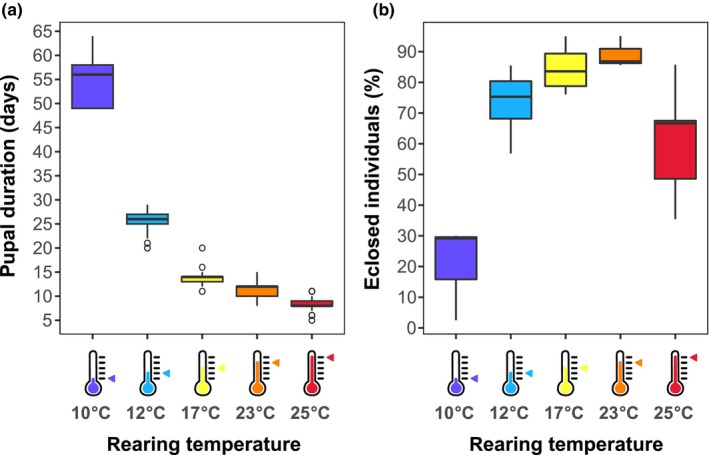
Rearing temperature affects pupal duration and eclosion rate. (a) Third instar *Eristalis tenax* larvae were moved to different temperatures just before pupation, and kept in that temperature until eclosion. The data show the duration of the pupal stage, as a function of pupal rearing temperature (for *N* numbers and statistical results, see Tables 1, 2, 3). (b) The data show the percentage of pupae that eclosed as a function of rearing temperature per batch.

To investigate the effect rearing temperature has on *E*. *tenax* hoverflies (Figure 1a), we exposed pupae to different ambient temperatures (Figure 1b). We found that rearing temperature had a significant effect on the length of the pupation period, that is, the time it took flies to emerge from the pupal cases (Figure 2a, ANOVA, *R* ^2^ = 88.44%, *p* < .001). The length of the pupation period was also slightly, though significantly, different between the batches (ANOVA, *R* ^2^ = 8.88%, *p* < .001). When the pupae were kept at 10°C, it took almost 2 months (54.5 ± 4.6 days) for them to eclose, whereas it took only 8.3 ± 1.3 days for them to eclose when kept at 25°C (Figure 2a).

We next looked at how many pupae eclosed after being exposed to the different temperatures. We found that an average 84.6 ± 8.4% of pupae eclosed successfully after being reared at 17°C (yellow data, Figure 2b), and a similar 89.2 ± 5.1% at 23°C. However, a rearing temperature of 12°C only yielded 73.2 ± 12.2% eclosion success, and 25°C even less with 60.4 ± 19.2%. The least pupae eclosed successfully when reared in 10°C, reaching only 20.6 ± 15.6% (Figure 2b). The differences between the rearing temperatures were significant (quasi‐binomial GLM, *p* < .001), whereas the batches did not differ significantly from each other (*p* = .470, Table 3).

**FIGURE 3:**
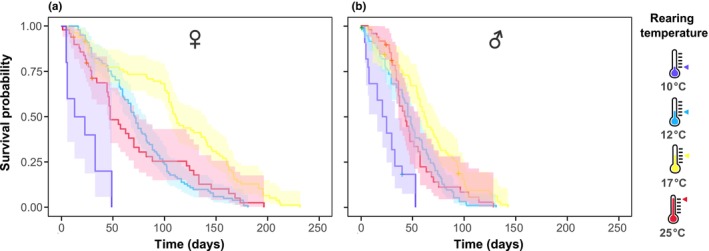
Rearing temperature affects survival. (a) A Kaplan–Meier plot of female *Eristalis tenax* survival, color coded according to rearing temperature (for *N* numbers and statistical results, see Tables 1, 2, 3). (b) Survival of male *Eristalis tenax*. In both panels, the data are shown as survival probability ± confidence intervals. Censored data points appear as plus signs (+).

Following eclosion, the survival rate of hoverflies reared at four different temperatures was tracked. We found that the rearing temperature significantly affected the adult life span (Cox proportional hazards model, *p* < .001, Table 3), with hoverflies reared at 17°C (yellow, Figure 3) living for longer than those reared 12°C (cyan, Figure 3) or 25°C (red, Figure 3). Strikingly, hoverflies reared at 10°C (purple, Figure 3) lived for a much shorter time than the hoverflies reared at the warmer temperatures. The survival rate also differed significantly between batches (Cox proportional hazards model, *p* < .001).

The differences in life span between individuals reared in different temperatures occurred both in female (Figure 3a) and male hoverflies (Figure 3b). However, female hoverflies lived much longer than males after rearing at any temperature (Cox proportional hazards model, *p* < .001), with the oldest female reaching an impressive age of 231 days (yellow, Figure 3a), much longer than previously recorded (Dolley Jr. & White, 1951; Gladis, 1994; Heal, 1989; Nicholas et al., 2018). Hoverflies reared at 10°C resulted in fewer adults eclosing (blue, Figure 2b) and poorer survival (blue, Figure 3). As this resulted in fewer numbers of adult hoverflies, they were excluded from further analyses. In addition, the hoverflies reared at 23°C (room temperature) were excluded from further analysis.

### 3.2 Hoverfly morphometrics are affected by rearing temperature

**FIGURE 4:**
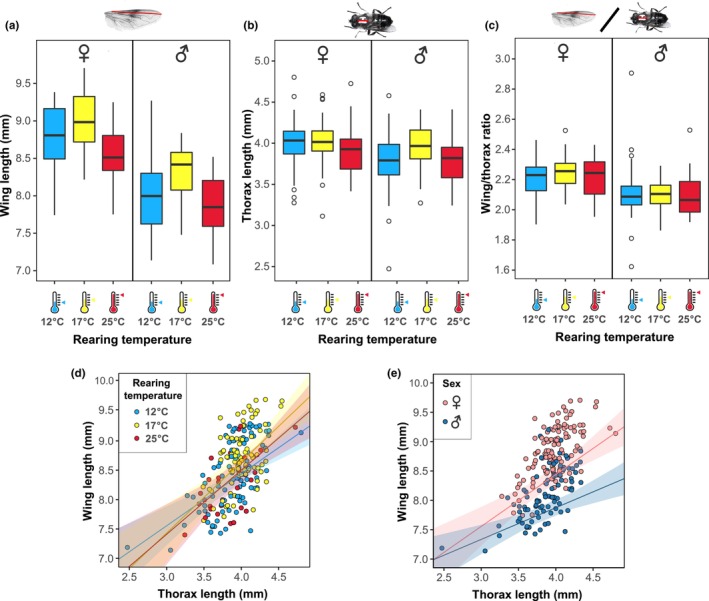
Rearing temperature affects wing length. (a) Hoverfly wing length as a function of rearing temperature (for *N* numbers and statistical results, see Tables 1, 2, 3). (b) Hoverfly thorax length as a function of rearing temperature. (c) Ratio between wing length and thorax length as a function of rearing temperature. (d) The wing length as a function of thorax length, color coded according to rearing temperature. Regression line with 95% confidence interval is shown for each rearing temperature group. (e) The thorax length as a function of wing length, color coded for males and females. Regression line with 95% confidence interval is shown for each sex. For statistical details, see Data S1.

Next, we quantified the size of the hoverflies. We found that wing and thorax length were both affected by sex and batch identity (ANOVA, *p* < .001, Figure 4a,b and Table 3). Wing length was significantly affected by the rearing temperature (ANOVA, *p* < .01, Figure 4a), with the longest wings in hoverflies reared at 17°C (yellow, Figure 4a), which were significantly longer than those of hoverflies reared at 12°C (Tukey's HSD, *p* < .001) and 25°C (Tukey's HSD, *p* < .05). However, there was no difference in wing length between hoverflies reared in 12°C or 25°C (Tukey's HSD, *p* = .305), and the overall effect of rearing temperature was small (*R* ^2^ = 1.53%). Females had longer wings than males (ANOVA, *R* ^2^ = 31.54%, *p* < .001) and the wing length differed between batches (ANOVA, *R* ^2^ = 36.29%, *p* < .001). The effect of rearing temperature on thorax length was not significant (ANOVA, *p* = .349, Figure 4b and Table 3). Nevertheless, we found that females had slightly longer thoraxes than males (ANOVA, *R* ^2^ = 3.57%, *p* < .001), and thorax length differed between batches (ANOVA, *R* ^2^ = 22.23%, *p* < .001).

When looking at the wing/thorax length ratio, we found that there was no difference between rearing temperatures (ANOVA, *p* = .544, Figure 4c) or batches (ANOVA, *p* = .43). However, females had a significantly larger wing/thorax ratio than males (ANOVA, *R* ^2^ = 13.9%, *p* < .001, Figure 4c). Furthermore, we analyzed differences in allometry by plotting the wing length as a function of thorax length. We found that hoverflies reared in different temperatures followed the same allometric relationship (LM, *p* = .116, Figure 4d). However, the allometric gradient was higher in females than in males (LM, *p* = .007, Figure 4e). Additionally, we found a significant difference in allometric gradients between batches (LM, *p* < .011).

**FIGURE 5:**
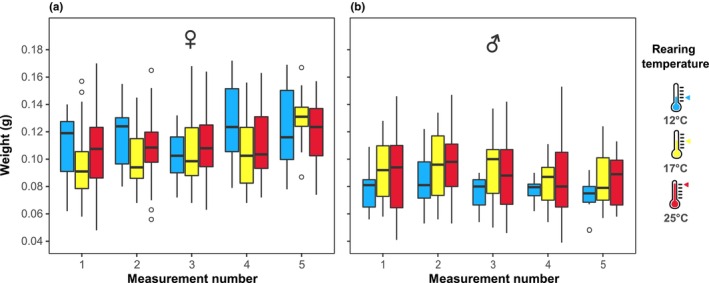
Females are heavier than males. (a) Female *Eristalis tenax* weight, color coded according to rearing temperature (for *N* numbers and statistical results, see Tables 1, 2, 3). (b) Male *Eristalis tenax* weight, color coded according to rearing temperature. Each measurement was taken 5–15 days apart.

We weighed the flies every 5–15 days until the time of death and found that females were heavier than males (LM, *R* ^2^ = 20.50%, *p* < .001, Figure 5). Additionally, we found a weak, though significant, interaction between sex and age (measurement number), implying that weight changes over time followed different trends in males and females (LM, *R* ^2^ = 1.58%, *p* = .003, Figure 5). Indeed, females slightly gained weight over time (Simple Slopes Analysis, −0.02 ± 0.01, *p* < .01), whereas males did not (Simple Slopes Analysis, *p* = .17). Nevertheless, neither the weight (LM, *p* = .711), nor its changes over time (LM, *p* = .430), were affected by the rearing temperature. The only exception were males reared in 17°C, which were slightly heavier than other males (EMM, *p* < .05), and thus, the interaction between sex and rearing temperature was significant (LM, *R* ^2^ = 2.19%, *p* = .002).

**FIGURE 6:**
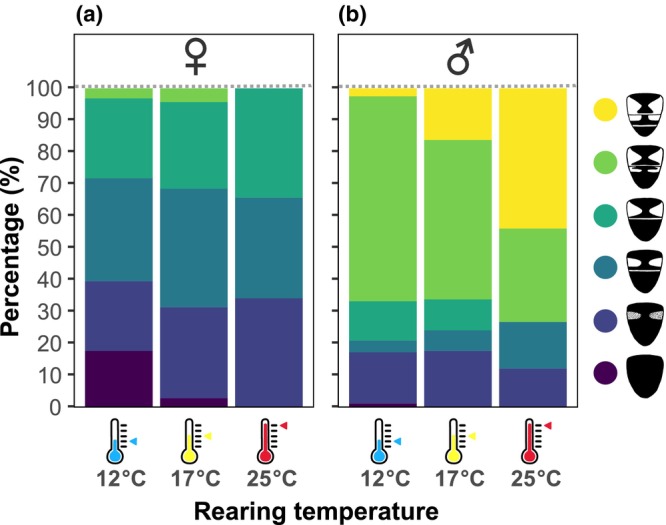
Females are darker than males. (a) Female *Eristalis tenax* abdominal coloration as a function of rearing temperature (for *N* numbers and statistical results, see Tables 1, 2, 3). (b) Male *Eristalis tenax* abdominal coloration as a function of rearing temperature. Color coding for abdominal coloration (after Heal, 1979) is given on the right.

As a final morphometric measure, we looked at abdominal coloration, classified into six categories according to Heal (1979). There was a weak trend in the extreme color categories (Figure 6), with “ultra‐light” and “light” coloration being seemingly more prevalent among males reared in high temperature (25°C) and “dark” coloration being seemingly more prevalent among females reared in low temperature (12°C). However, other color categories (“medium light,” “medium,” and “medium dark”) showed no such trend, and thus, the overall effect of rearing temperature on abdominal coloration was not significant (Cumulative Link Model, *p* = .326). Nevertheless, we found that females (Figure 6a) were overall darker than males (Figure 6b, CLM, *p* < .001, see also Figure 1a) and that abdominal coloration differed significantly between batches (CLM, *p* < .001).

### 3.3 Locomotor activity decreases with temperature

**FIGURE 7:**
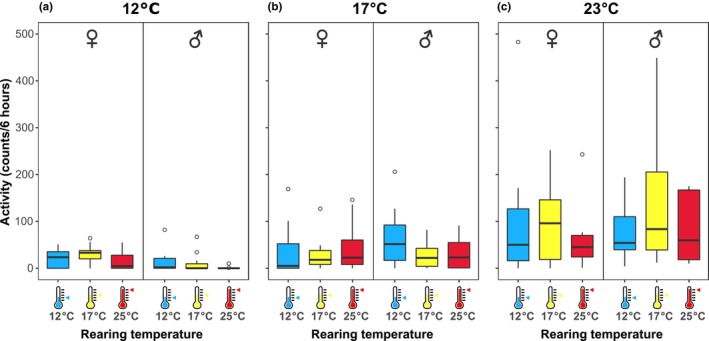
Ambient temperature affects locomotor activity. (a) Hoverfly locomotor activity in 12°C as a function of pupal rearing temperature, with females on the left (for *N* numbers and statistical results, see Tables 1, 2, 3) and males on the right. (b) Hoverfly locomotor activity in 17°C as a function of rearing temperature. (c) Hoverfly locomotor activity at 23°C as a function of rearing temperature.

As a measure of fitness, we quantified hoverfly physical activity, using a locomotor activity monitoring system (LAMS). We found that the activity of the hoverflies increased with testing temperature (GLM, *p* < .001, Table 3). Males and females were in general equally active (GLM, *p* = .949, Figure 7), with highest level of activity when tested at 23°C (Figure 7c). The activity of males decreased when the ambient temperature was 12°C (Figure 7a) compared to their activity in 17°C (Figure 7b, Tukey's HSD, *p* < .001). This was not observed in females (Tukey's HSD, *p* = .63). We found no significant effect of rearing temperature (GLM, *p* = .404) or batch identity (GLM, *p* = .423). Notably, when we tested if the hoverflies were more active in the temperatures in which they were reared as pupae (interaction between rearing and testing temperature), we found only a weak, borderline‐significant effect (GLM, *p* = .058, *F* = 2.32).

## 4 DISCUSSION

We found that ambient temperature during the *E*. *tenax* pupal stage (Figure 1) had a strong effect on eclosion timing and developmental rates, as well as the wing length and lifespan, of adult hoverflies. We found that higher rearing temperature led to shorter pupal duration (Figure 2a). In addition, pupal rearing at 17°C gave higher eclosion rates (Figure 2b) of hoverflies that lived longer (Figure 3) and had longer wings (Figure 4), compared with either higher or lower rearing temperatures. However, we found no significant effect of rearing temperature on weight (Figure 5), coloration (Figure 6) or activity (Figure 7), even though we found strong sexual dimorphism in these traits (Table 3).

### 4.1 Pupal development

We exposed hoverfly pupae to different ambient temperatures. Earlier work in butterflies and moths have shown similar thermal effects whether the animals were exposed at the egg, larval, or pupal stage (Kingsolver et al., 2011), suggesting that our results may be relevant to other developmental stages. We found that the duration of the pupal stage decreased substantially with temperature (Figure 2a), consistent with previous work on *E*. *tenax* (Heal, 1989; Ottenheim et al., 1996), *Eristalis arbustorum* (Ottenheim & Holloway, 1995), *Episyrphus balteatus* (Hart et al., 1997) and other insects (e.g. Kuntz & Eisen, 2014; Li et al., 2013). We hypothesized that developmental rate would follow a typical, skewed TPC, as seen in, for example, sepsid flies (Khelifa et al., 2019). Indeed, we found that the eclosion rate was optimal at 17°C and 23°C, but decreased substantially when the pupae were reared closer to either the lower or upper thermal limits, i.e. when reared at 10°C or 25°C (Figure 2b). This is consistent with *Eristalis arbustorum*, which have higher pupal mortality at 8°C than warmer rearing temperatures (Ottenheim et al., 1996). However, emergence rates showed less clear dependence on temperature in a previous study on *E*. *tenax* (Heal, 1989). It is possible that other factors contribute to different results between studies, such as the larval environment. For example, here, *E*. *tenax* larvae were reared in fresh rabbit dung (as in Nicholas et al., 2018), whereas the previous studies used dried rabbit dung collected from sand dunes (Heal, 1989; Ottenheim & Holloway, 1995). As the larvae may feed on microorganisms within the rabbit dung (Ottenheim & Holloway, 1995), their food source during the larval stage could have affected future development. Indeed, in blowflies, the conditions during the larval stage, including larval crowding, have a large effect on development and subsequent size and survival of the adult flies (Ireland & Turner, 2006). Additionally, the ability to adapt to the developmental temperature are partly heritable in *E*. *tenax*, and the Australian population that we studied might be adapted to a different range of temperatures than the previously studied British population (Heal, 1989). Despite this, batches did not differ significantly in eclosion rate in our study (Table 3), which may indicate that environmental factors probably played a more significant role than genetic ones.

### 4.2 Hoverfly fitness

The hoverflies reared at 17°C lived substantially longer than those reared at other temperatures (Figure 3), as hypothesized. Similar survival TPCs have been measured in, for example, mosquitoes (Paaijmans et al., 2013). Furthermore, we hypothesized that hoverflies would have higher locomotor activity at the temperature they were exposed to during development, as predicted by the beneficial acclimation hypothesis. We did not find a significant interaction between rearing and testing temperature (*p* = .596). In contrast, water beetles exposed to low temperatures for 5 days were more active at higher test temperatures than those exposed to high temperatures (Pallarés et al., 2021).

As expected (Angilletta et al., 2002; Berrigan & Partridge, 1997), we found that activity increased substantially with ambient temperature (Figure 7), which is consistent with field studies, showing lower hoverfly activity at lower air temperatures. Indeed, it has been suggested that thermal economy is likely the main determinant in structuring hoverfly diurnal activity (Gilbert, 1985). In northern Europe, the temperature differences during the day, from the first hoverfly sightings around 8:00, to their disappearance after 18:00 (Ottenheim, 2000), are substantial. Indeed, the first *E*. *tenax* observations (Cambridge, UK) take place when the air temperature is 10°C, and the first moving animals are observed when air temperature reaches 11°C (Gilbert, 1985). However, *E*. *tenax* are often inactive or cleaning below 13°C (Gilbert, 1985), which is consistent with the low locomotor activity that we recorded at 12°C (Figure 7a). In the field, *E*. *tenax* start feeding and flying between flowers when the temperature is 13°C–21°C (Gilbert, 1985). Indeed, we saw much higher activity at 17°C and at 23°C (Figure 7b,c).

In *Musca domestica*, activity increases with temperature, until about 30°C when it decreases again (Schou et al., 2013). This suggests that if we had tested locomotor activity (Figure 7) at even higher temperatures, their activity would likely have decreased. Indeed, *E*. *tenax* hoverflies are usually phototactic, but if the ambient temperature is above 40°C, they instead become photophobic (Dolley Jr. & Golden, 1947) and temperatures over 50°C are lethal (Dolley Jr. & White, 1951).

### 4.3 Wing length

We hypothesized that colder temperatures would result in larger animals, as expected from the temperature–size rule (Atkinson, 1994). However, we saw no significant effect of rearing temperature on body length (Figure 4b and Table 3) or weight (Figure 5 and Table 3), but instead saw the longest wings in hoverflies reared at 17°C, significantly different from colder (12°C) and warmer (25°C) rearing temperatures, in both males and females (Figure 4a). However, the allometric relationship between wing and thorax length was not affected by rearing temperature (Figure 4c,d).

In *Eristalis arbustorum*, there is also a clear link between rearing temperature and wing length, with longer wings after rearing at warmer temperatures (20°C compared to 12°C, Ottenheim & Volmer, 1999). In butterflies, wing‐length is a good predictor of dispersal ability (Sekar, 2012), whereas in damselflies wing shape, but not wing length, is affected by rearing temperature, and this affects future flight performance (Arambourou et al., 2017). Previous work showed a seasonal variation in wing shape, but not wing size, in *E*. *tenax* (Francuski et al., 2011). This has been interpreted as a functional adaptation to different behavioral requirements during different parts of the season.

Hoverflies disperse after eclosion and many are long distance migrators (Menz et al., 2019). If wing‐length is correlated with dispersal and migration in hoverflies, then reduced wing‐length after development outside the *T*_opt_ will negatively affect this ability. For wild hoverflies, this could be a disadvantage since it will reduce their ability to move to new areas and thus reduce genetic interchange between isolated populations. Long‐term, this could have negative impacts on the abundance and fitness of the hoverflies, and hence on their pollination ability. However, if hoverflies are to be reared as commercial pollinators, reduced dispersal via reduced wing‐length could be exploited to ensure that they remain within the area they are needed.

### 4.4 Coloration

We hypothesized that pupae reared at lower temperatures would be darker, according to the thermal melanism hypothesis. Abdominal coloration in *E*. *tenax* varies substantially between individuals, but it was not significantly affected by rearing temperature in our study (Figure 6 and Table 3). Previous work showed that colder pupal rearing lead to darker coloration in some *Eristalis* species, ranging from a weak trend (Heal, 1989) to a significant effect (Ottenheim et al., 1996), especially in males. Since colder temperatures lead to longer developmental times, there could be a correlation between development time and coloration rather than the temperature per se (Ottenheim et al., 1996). Since lower temperature during the pupal stage gives darker abdominal patterns (Heal, 1989) darker forms are more common in the cooler spring months and brighter forms are more common in the warmer summer months (Holloway, 1993). This has been suggested to be functional for thermoregulation as darker colors absorb more light, and will warm up the animal, which is more important during the cooler spring (Holloway, 1993), as seen in other insects (Forsman, 2011). Indeed, during cool days *E*. *tenax* are significantly more likely to be seen in the sun than in the shade, showing a need for solar warming (Gilbert, 1985). However, later studies have failed to find seasonal changes in coloration, showing instead that males retain their mostly bright, yellow abdominal coloration (Figure 1a) throughout the year (Francuski et al., 2011).

It has been suggested that high temperature during the pupal stage (but not larval stage) suppresses melanin production, which leads to adults with a lighter abdominal coloration (Heal, 1989). However, controlled breeding experiments indicate that coloration is not only under environmental control but also under genetic control, and that there is a dominant allele for the light phenotype (Heal, 1979). Indeed, we saw that coloration depended strongly on batch (ANOVA, *p* < .001), but not on the rearing temperature (ANOVA, *p* = .326, Figure 6), supporting genetic control of coloration over environmental.

Males were significantly brighter than females (Figures 1a and 6 and Table 3), consistent with previous results (Francuski et al., 2011; Ottenheim et al., 1996). Indeed, across the *Eristalis* genus, females are often darker than the males (Holloway, 1993). Abdominal coloration likely reflects a balance between requirements for thermoregulation and protection against predators (Francuski et al., 2011; Heal, 1989; Ottenheim et al., 1996). *Eristalis tenax* are Batesian mimics of hymenopterans (Atkins, 1948), and darker honeybee‐like color (Figure 6a) might help females deceive birds more effectively. Additionally, dark color might make the females less conspicuous against a dark background during resting. Indeed, *E*. *tenax* females fly predominantly in the shade, whereas male *E*. *tenax* fly equally in sun and shade (Gilbert, 1985). On the contrary, light coloration of males could contribute to efficient thermoregulation during their courtship flights (Gilbert, 1984).

### 4.5 Sexual dimorphism

Besides the coloration (Figure 6), we found that several traits were strongly sexually dimorphic. For example, females had longer wings (Figure 4a), longer thorax (Figure 4b), steeper allometric gradient between wing length and thorax length (Figure 4e), and were heavier (Figure 5). However, as we did not exclude flies that died during the experimental period, apparent weight increase of our females could have been affected or partly caused also by possible longer lifespan of heavier hoverflies. Nevertheless, previous work showed that females gain more weight over time (Nicholas et al., 2018) and that female wings are larger than male wings in *E*. *tenax* (Francuski et al., 2011, 2013). The larger size is likely associated with females reaching sexual maturity and storing eggs. However, in another hoverfly, the marmalade hoverfly *Episyrphus balteatus*, males are heavier than females, and weight decreases substantially with rearing temperature (Hart et al., 1997).

The most striking sexual dimorphism was that females lived much longer than males, after pupal rearing at any temperature (Figure 3). This long survival is especially striking compared with previous accounts, which showed room temperature survival of 2–3 months (Nicholas et al., 2018), 77 days (Dolley Jr. & White, 1951), 4 months (Gladis, 1994) or 18 weeks (Heal, 1989). That females live longer than males could have a functional significance. Indeed, in the Northern hemisphere, in late autumn mated females will overwinter. They will store the sperm and undeveloped eggs, then lay fertilized eggs in early spring to start the new generation (Kendall & Stradling, 1972). Being able to survive for many months would support this amazing ability to overwinter.

### 4.6 Pollination service

Pollination is a key ecosystem service with 75% of our crops benefitting directly from animal pollination (Doyle et al., 2020). Hoverflies, including *E*. *tenax*, are excellent pollinators, maybe even as good as bees (Gladis, 1997). With honeybee colonies being threatened by *Varroa*, there is a growing interest in using non‐bee pollinators in crop production, and then especially hoverflies (Doyle et al., 2020), which are identified as key species for mass rearing for pollination services (Cook et al., 2020). Sadly, a loss of habitat associated with increased agricultural land use leads to loss of wild, native pollinators (Sánchez‐Bayo & Wyckhuys, 2019). Exotic pollinators, like *E*. *tenax* in New Zealand, could then become more important as they can adapt to human‐modified environments (Stavert et al., 2018). Furthermore, flies often tend to be more resilient than bees to land use change (Rader et al., 2020). Indeed, around some agricultural areas, hoverflies are often more abundant than all bee species (see e.g. Doyle et al., 2020). By understanding more about the development, and subsequent adult traits of hoverflies, we here provide data that could be used for e.g. increasing the use of hoverflies is crop pollination.

## AUTHOR CONTRIBUTIONS

**Klára Daňková:** Conceptualization (lead); data curation (lead); formal analysis (equal); investigation (lead); methodology (equal); software (equal); validation (equal); visualization (lead); writing – original draft (lead); writing – review and editing (equal). **Sarah Nicholas:** Conceptualization (supporting); investigation (equal); methodology (equal); supervision (equal); writing – review and editing (equal). **Karin Nordström:** Conceptualization (equal); data curation (equal); funding acquisition (lead); methodology (equal); project administration (equal); resources (lead); writing – review and editing (lead).

## FUNDING INFORMATION

This research was funded by the US Air Force Office of Scientific Research (AFOSR, FA9550‐19‐1‐0294), the Australian Research Council (ARC, FT180100289, DP210100740, DP230100006), Grant Agency of Charles University (464220/2020), the Faculty of Science Foundation of Charles University, and the Mobility Fund of Charles University.

## CONFLICT OF INTEREST STATEMENT

The authors declare no conflict of interest.

## Supporting information


Data S1:

## Data Availability

All data have been submitted to DataDryad. https://doi.org/10.5061/dryad.2z34tmpr1.
