# Supplementary material for: Temperature during pupal development affects hoverfly developmental time, adult life span, and wing length
Source: Ecol Evol. 2023 Oct 24;13(10):e10516. doi: 10.1002/ece3.10516 (PMC10597744; doi:10.1002/ece3.10516)
Supplement: Supplementary file 1 — Data S1: [file ECE3-13-e10516-s001.docx]

***Supplementary information for***

**Temperature during pupal development affects hoverfly developmental time, adult lifespan and wing length**

*Klára Daňková^1, 2^, Sarah Nicholas^1^ & Karin Nordström^1,3^*

This supplementary information provides a summary of the output of each statistical analysis. Each heading describes the data set analysed, what statistical test was performed, and the figure that shows the data. The blue text under each heading shows the R commands used, and the table the output of the statistical analysis. In all cases, the significance codes are the following: ‘***’ for p < 0.001; ‘**’ for p < 0.01; ‘*’ for p < 0.05; ‘.’ for p < 0.1; ‘ ’ for p < 1.

1. **Pupal duration, ANOVA, Figure 2a**

> model_pupalD<-aov(sqrt(Pupal.duration)~Batch.ID+Rearing.temperature,data=pupalD)

> anova(model_pupalD)

Analysis of Variance Table

Response: sqrt(Pupal.duration)

|  | Df | Sum Sq | Mean Sq | F value | Pr(>F) | Sign. code |
| --- | --- | --- | --- | --- | --- | --- |
| Batch.ID | 6 | 77.58 | 12.929 | 417.02 | < 2.2e-16 | *** |
| Rearing.temp | 4 | 611.85 | 152.961 | 4933.55 | < 2.2e-16 | *** |
| Residuals | 793 | 24.59 | 0.031 |  |  |  |

1. **Percentage of successfully eclosed individuals, GLM, Figure 2b**

> model_eclosion_rate<-glm(cbind(Eclosed,Not.Eclosed)~Batch.ID+Rearing.temperature, family="quasibinomial", data=eclosionR)

> anova(model_eclosion_rate,test="F")

Analysis of Deviance Table

Model: quasibinomial, link: logit

Response: cbind(Eclosed, Not.Eclosed)

Terms added sequentially (first to last)

|  | Df | Deviance | Resid. Df | Resid. Dev | F | Pr(>F) | Sign. code |
| --- | --- | --- | --- | --- | --- | --- | --- |
| NULL |  |  | 18 | 289.39 |  |  |  |
| Batch.ID | 6 | 23.51 | 12 | 265.88 | 1.0299 | 0.4704105 |  |
| Rearing.temp | 4 | 233.96 | 8 | 31.92 | 15.3736 | 0.0007972 | *** |

1. **Survival: Cox proportional hazards model, Figure 3**

> #Analyzing the effect of explaining variables via model comparison

> anova(model_survival_all,model_survival_sex)

Analysis of Deviance Table

Cox model: response is Surv(Lifespan, Status)

|  | loglik | Chisq | Df | Pr(>F) | Sign. code |
| --- | --- | --- | --- | --- | --- |
| Batch.ID:Sex:Rearing.temp | -2050.3 |  |  |  |  |
| Batch.ID:Rearing.temp | -2097.6 | 94.592 | 1 | < 2.2e-16 | *** |

> anova(model_survival_all,model_survival_temp)

Analysis of Deviance Table

Cox model: response is Surv(Lifespan, Status)

|  | loglik | Chisq | Df | Pr(>F) | Sign. code |
| --- | --- | --- | --- | --- | --- |
| Batch.ID:Sex:Rearing.temp | -2050.3 |  |  |  |  |
| Batch.ID:Sex | -2076.8 | 52.97 | 2 | 3.145e-12 | *** |

> anova(model_survival_all,model_survival_batch)

Analysis of Deviance Table

Cox model: response is Surv(Lifespan, Status)

|  | loglik | Chisq | Df | Pr(>F) | Sign. code |
| --- | --- | --- | --- | --- | --- |
| Batch.ID:Sex:Rearing.temp | -2050.3 |  |  |  |  |
| Sex:Rearing.temp | -2077.2 | 53.864 | 4 | 5.621e-11 | *** |

1. **Wing length, ANOVA, Figure 4a**

> model_wings<-aov(sqrt(Wing.length)~Batch.ID+Sex+Rearing.temperature, data=wings)

> anova(model_wings)

Analysis of Variance Table

Response: sqrt(Wing.length)

|  | Df | Sum Sq | Mean Sq | F value | Pr(>F) | Sign. code |
| --- | --- | --- | --- | --- | --- | --- |
| Batch.ID | 3 | 0.70905 | 0.23635 | 78.919 | < 2.2e-16 | *** |
| Sex | 1 | 0.61616 | 0.61616 | 205.739 | < 2.2e-16 | *** |
| Rearing.temp | 2 | 0.08626 | 0.04313 | 14.401 | 1.571e-06 | *** |
| Residuals | 181 | 0.54207 | 0.00299 |  |  |  |

1. **Thorax length, ANOVA, Figure 4b**

> model_thorax<-aov(Thorax.length~Camera+Alive.dead+Batch.ID+Sex+Rearing.temperature, data=thorax)

> anova(model_thorax)

Analysis of Variance Table

Response: Thorax.length

|  | Df | Sum Sq | Mean Sq | F value | Pr(>F) | Sign. code |
| --- | --- | --- | --- | --- | --- | --- |
| Camera | 1 | 1.8610 | 1.86095 | 30.1681 | 9.097e-08 | *** |
| Alive.dead | 1 | 0.3433 | 0.34328 | 5.5649 | 0.0190311 | * |
| Batch.ID | 3 | 5.7273 | 1.90911 | 30.9488 | < 2.2e-16 | *** |
| Sex | 1 | 0.9190 | 0.91901 | 14.8982 | 0.0001418 | *** |
| Rearing.temp | 2 | 0.1303 | 0.06515 | 1.0561 | 0.3492145 | *** |
| Residuals | 272 | 16.7786 | 0.06169 |  |  |  |

1. **Wing/thorax ratio, ANOVA, Figure 4c**

> model_ratio<-aov(Wing.thorax.ratio~Camera+Alive.dead+Batch.ID+Sex+Rearing.temperature, data=ratio)

> anova(model_ratio)

Analysis of Variance Table

Response: Wing.thorax.ratio

|  | Df | Sum Sq | Mean Sq | F value | Pr(>F) | Sign. code |
| --- | --- | --- | --- | --- | --- | --- |
| Camera | 1 | 0.0313 | 0.03127 | 1.7428 | 0.18830 |  |
| Alive.dead | 1 | 0.1164 | 0.11644 | 6.4887 | 0.01161 | * |
| Batch.ID | 3 | 0.0827 | 0.02755 | 1.5355 | 0.20652 |  |
| Sex | 1 | 0.5373 | 0.53725 | 29.9381 | 1.325e-07 | *** |
| Rearing.temp | 2 | 0.0219 | 0.01094 | 0.6095 | 0.54462 |  |
| Residuals | 200 | 3.5891 | 0.01795 |  |  |  |

1. **Wing allometry, LM, Figure 4d and 4e**

> model_allometry<-lm(Wing.length~Thorax.length*(Batch.ID+Sex+Rearing.temperature+Thorax.length),data=wings)

> summary (model_allometry)

Residuals:

Min 1Q Median 3Q Max

-0.9175 -0.1281 0.0090 0.1323 0.7297

|  | Df | Sum Sq | Mean Sq | F value | Pr(>F) | Sign. code |
| --- | --- | --- | --- | --- | --- | --- |
| Thorax.length | 1 | 21.7598 | 21.7598 | 330.8505 | < 2.2e-16 | *** |
| Batch.ID | 3 | 5.5931 | 1.8644 | 28.3471 | 1.29E-14 | *** |
| Sex | 1 | 13.843 | 13.843 | 210.4789 | < 2.2e-16 | *** |
| Rearing.temp | 2 | 1.9968 | 0.9984 | 15.1801 | 9.76E-07 | *** |
| Thorax.length:Batch.ID | 3 | 0.76 | 0.2533 | 3.8518 | 0.010823 | * |
| Thorax.length:Sex | 1 | 0.4874 | 0.4874 | 7.4102 | 0.007244 | ** |
| Thorax.length:Rearing.temp | 2 | 0.2877 | 0.1438 | 2.1871 | 0.115756 |  |
| Residuals | 152 | 9.9969 | 0.0658 |  |  |  |

> anova(model_allometry)

Analysis of Variance Table

Response: Wing.length

Residuals:

Min 1Q Median 3Q Max

-0.9175 -0.1281 0.0090 0.1323 0.7297

|  | Estimate | Std. Error | t value | Pr(>\|t\|) | Sign. code |
| --- | --- | --- | --- | --- | --- |
| (Intercept) | 3.8111 | 0.7649 | 4.982 | 1.69E-06 | *** |
| Thorax.length | 1.2375 | 0.2007 | 6.166 | 6.04E-09 | *** |
| Batch.ID5 | 3.4693 | 1.3889 | 2.498 | 0.0136 | * |
| Batch.ID7 | 4.5249 | 0.9637 | 4.695 | 5.90E-06 | *** |
| Batch.IDweight | 1.2653 | 0.889 | 1.423 | 0.1567 |  |
| SexM | 0.9656 | 0.6264 | 1.541 | 0.1253 |  |
| Rearing.temp.L | -0.6055 | 0.6945 | -0.872 | 0.3847 |  |
| Rearing.temp.Q | 0.663 | 0.6744 | 0.983 | 0.3271 |  |
| Thorax.length:Batch.ID5 | -0.8191 | 0.3495 | -2.344 | 0.0204 | * |
| Thorax.length:Batch.ID7 | -1.0199 | 0.2453 | -4.157 | 5.36E-05 | *** |
| Thorax.length:Batch.IDweight | -0.3413 | 0.2336 | -1.461 | 0.146 |  |
| Thorax.length:SexM | -0.4025 | 0.1585 | -2.539 | 0.0121 | * |
| Thorax.length:Rearing.temp.L | 0.1702 | 0.1802 | 0.944 | 0.3464 |  |
| Thorax.length:Rearing.temp.Q | -0.2106 | 0.1698 | -1.241 | 0.2166 |  |

Residual standard error: 0.2565 on 152 degrees of freedom

Multiple R-squared: 0.8173, Adjusted R-squared: 0.8017

F-statistic: 52.31 on 13 and 152 DF, p-value: < 2.2e-16

1. **Weight gain, LM, Figure 5**

> model_weight<-lm(log(Weight)~(Measurement.number+Sex+Rearing.temperature)^2,data=weight_gain)

> summary(model_weight)

Residuals:

Min 1Q Median 3Q Max

-0.75053 -0.19155 0.01952 0.18695 0.59488

| Coefficients: | Estimate | Std. Error | t value | Pr(>\|t\|) | Sign. code |
| --- | --- | --- | --- | --- | --- |
| (Intercept) | -2.332483 | 0.038488 | -60.603 | < 2e-16 | *** |
| Measurement.number | 0.035076 | 0.012249 | 2.864 | 0.00439 | ** |
| SexM | -0.126782 | 0.055413 | -2.288 | 0.022616 | * |
| Rearing.temp.L | -0.029307 | 0.054882 | -0.534 | 0.593609 |  |
| Rearing.temp.Q | 0.101729 | 0.051315 | 1.982 | 0.048054 | * |
| Measurement.number:SexM | -0.053374 | 0.017957 | -2.972 | 0.003119 | ** |
| Measurement.number:Rearing.temp.L | -0.001779 | 0.015903 | -0.112 | 0.91097 |  |
| Measurement.number:Rearing.temp.Q | -0.019991 | 0.015454 | -1.294 | 0.196506 |  |
| SexM:Rearing.temp.L | 0.058545 | 0.043413 | 1.349 | 0.178176 |  |
| SexM:Rearing.temp.Q | -0.146253 | 0.042603 | -3.433 | 0.000654 | *** |

Residual standard error: 0.2585 on 438 degrees of freedom

Multiple R-squared: 0.252, Adjusted R-squared: 0.2366

F-statistic: 16.4 on 9 and 438 DF, p-value: < 2.2e-16

> anova(model_weight)

Analysis of Variance Table

Response: log(Weight)

|  | Df | Sum Sq | Mean Sq | F value | Pr(>F) | Sign. code |
| --- | --- | --- | --- | --- | --- | --- |
| Measurement.number | 1 | 0.2062 | 0.2062 | 3.0847 | 0.079729 | . |
| Sex | 1 | 8.0251 | 8.0251 | 120.056 | < 2.2e-16 | *** |
| Rearing.temp | 2 | 0.0457 | 0.0228 | 0.3418 | 0.710694 |  |
| Measurement.number:Sex | 1 | 0.6176 | 0.6176 | 9.2392 | 0.002511 | ** |
| Measurement.number:Rearing.temp | 2 | 0.113 | 0.0565 | 0.8449 | 0.430294 |  |
| Sex:Rearing.temp | 2 | 0.857 | 0.4285 | 6.4104 | 0.001803 | ** |
| Residuals | 438 | 29.2781 | 0.0668 |  |  |  |

> sim_slopes(model=model_weight, pred=Measurement.number, modx = Sex, john=F)

*Slope of Measurement.number when Sex = M:*

Est. S.E. t val. p

------- ------ -------- ------

-0.02 0.01 -1.38 0.17

*Slope of Measurement.number when Sex = F:*

Est. S.E. t val. p

------ ------ -------- ------

0.04 0.01 2.86 0.00

1. **Coloration, cumulative link model (CLM), Figure 6**

> model_coloration_test1<-clm(Coloration~Alive.dead+Camera+Photo.age+Batch.ID+Sex*Rearing.temperature,data=coloration)

> anova(model_coloration_test1)

Type I Analysis of Deviance Table with Wald chi-square tests

|  | Df | Chisq | Pr(>F) | Sign. code |
| --- | --- | --- | --- | --- |
| Alive.dead | 1 | 31.9913 | 1.549e-08 | *** |
| Camera |  |  |  |  |
| Photo.age | 1 | 7.3064 | 0.006871 | ** |
| Batch.ID | 4 | 42.8859 | 1.093e-08 | *** |
| Sex | 1 | 110.5134 | < 2.2e-16 | *** |
| Rearing.temp | 2 | 2.2393 | 0.326398 |  |
| Sex:Rearing.temp | 2 | 2.4806 | 0.289295 |  |

1. **Activity, GLM, Figure 7**

> model_activity_all_1<-glm(sqrt(Activity.counts)~Testing.temperature+Batch.ID+Sex+

+ Rearing.temperature+

+ Sex:Testing.temperature+

+ Rearing.temperature:Testing.temperature,

+ data=activity1, family="quasipoisson")

> anova(model_activity_all_1,test="F")

> anova(model_activity_all_1,test="F")

Analysis of Deviance Table

Model: quasipoisson, link: log

Response: sqrt(Activity.counts)

Terms added sequentially (first to last)

|  | Df | Deviance | Resid. Df | Resid. Dev | F | Pr(>F) | Sign. code |
| --- | --- | --- | --- | --- | --- | --- | --- |
| NULL |  |  | 203 | 874.13 |  |  |  |
| Testing.temp | 1 | 198.864 | 202 | 675.26 | 71.4447 | 7.047e-15 | *** |
| Batch.ID | 4 | 20.440 | 198 | 654.82 | 1.8358 | 0.12356 |  |
| Sex | 1 | 0.028 | 197 | 654.79 | 0.0102 | 0.91954 |  |
| Rearing.temp | 2 | 5.051 | 195 | 649.74 | 0.9073 | 0.40535 |  |
| Testing.temp:Sex | 1 | 18.369 | 194 | 631.37 | 6.5994 | 0.01096 | * |
| Testing.temp:Sex:Rearing.temp | 2 | 2.894 | 192 | 628.48 | 0.5198 | 0.59547 |  |
